# A bovine viral diarrhea virus type 1a strain in China: isolation, identification, and experimental infection in calves

**DOI:** 10.1186/1743-422X-11-8

**Published:** 2014-01-20

**Authors:** Wei Wang, Xinchuan Shi, Qin Tong, Yongwang Wu, Ming Qi Xia, Ye Ji, Wenzhi Xue, Hua Wu

**Affiliations:** 1 Institute of Special Economic Animal and Plant Science, CAAS, No. 4899, Juye Street, 130122 Changchun, PR China; 2 Sinovet (Beijing) Biotechnology Co., Ltd, No.5 Kaituo Street, Haidian District, 100085 Beijing, PR China; 3 MSD Animal Health, No. 10 Jiuxianqiao Road, Chaoyang District, 100015 Beijing, PR China

**Keywords:** Bovine viral diarrhea virus, BVDV, Cattle, Phylogenetic analysis, Pathogenesis, China

## Abstract

**Background:**

Bovine viral diarrhea virus (BVDV) is one of the most important pathogens in cattle. Previously, BVDV sub-genotypes of 1b, 1c, 1d, and 1 m were detected in China. However, isolation of BVDV type 1a from cattle has not been reported in China. In 2010, twenty nasal swabs and blood samples were collected from the cattle suspected BVDV infection in Henan province, China. A BVDV isolate was isolated using cell culture, and the pathogenesis of the virus isolate was studied.

**Methods:**

Virus isolation was performed on MDBK cells. The virus identification was conducted by RT-PCR, neutralization test and immunofluorescence assay. In order to determine the genotype of the newly isolated virus, the 5′ un-translated region (5′UTR) of the virus isolate was cloned, sequenced and phylogenetically analyzed. To evaluate the virulence of the virus isolate, four BVDV sero-negative calves were intranasally inoculated with the virus suspension. Rectal temperatures and clinical signs were recorded daily. Blood samples were analyzed for changes in white blood cell counts, and tissue samples were taken for histopathology analysis.

**Results:**

A new isolate of bovine viral diarrhea virus (BVDV), named HN01, was isolated from the nasal swabs using MDBK cell culture. The HN01 strain caused cytopathic effect (CPE) in MDBK cell cultures after two passages. The virus specifically reacted to BVDV1-specific monoclonal antibody in an immunofluorescence assay. A fragment of 288 bp of genome from this isolate was amplified by the RT-PCR. Phylogenetic analysis of 5′UTR indicated that the virus was BVDV 1a. In the pathogenesis study, four calves experimentally infected with the BVDV strain developed depression, cough and other clinical signs. Calves showed high temperature over 40°C, and white blood cell counts dropped more than 40%.

**Conclusions:**

A new subgenotype 1a strain of BVDV was firstly isolated from dairy cattle in China. The experimental infection showed that the virus was moderate pathogenic to cattle and can be used as a BVDV challenge virus to evaluate the efficacy of BVDV vaccines in the target animals.

## Background

Bovine viral diarrhea virus (BVDV) is a great economically pathogen in cattle and other ruminants in the world [1-10]. The virus is associated with several clinical symptoms, including diarrhea, respiratory disease, congenital malformations, reproductive disorders and mucosal disease [9,11-14].

BVDV belongs to the genus pestivirus with classical swine fever virus and border disease virus in the family *Flaviviridae*. The genome of the BVDV consists of a single positive-stranded RNA, which usually have a length of 12.3 kb [15]. Two biotypes of BVDV categorized as cytopathogenic or noncytopathogenic based on their activity in cell culture have been recognized in the past years [16,17]. Based on the basis of the nucleotide sequence of 5′-untranslated region (5′UTR), N^pro^ or E2 gene, BVDV strains can be divided into two different genotypes, BVDV1 and BVDV2 [18]. Each genotype can be further divided into different subgroups, and currently at least 11 genetic subgroups of BVDV1 and three genetic subgroups of BVDV2 are identified [3,6,18-24]. Recently, a new virus referred to as HoBi-like BVDV3 was identified in Europe, the virus can be divided into two sub-groups, Thai origin and Brazilian origin [25]. BVDV1 spreads worldwide in cattle population [22,23,26,27]. In the case of BVDV2 species, the highest occurrence is reported in the USA and Canada [25,28], partially in Japan [29-31], Indian [3], South America [19], and occasionally in some European countries [6,15,32-35].

Virulence is both important to understanding the mechanisms of pathology and selecting the challenge strains for evaluation of a vaccine. Variation in virulence among BVDV2 strains has been extensively reported [36-38], but much less information is available on variation in virulence among BVDV1 strains. To date, the strain NY-1 has been used as a challenge strain for evaluating efficacy of vaccine protection against BVDV1. While it is well characterized, the clinical presentation infected with NY-1 indicates it is more likely a low virulence strain [39]. So investigating an efficacious challenge virus to access the vaccine efficacy is very important.

To the present, many subgenotypes of BVDV1 have been isolated and detected in China [27,40,41]. Based on the phylogenetic tree, the clustering of BVDV1b and BVDV 1 m were the major prevalent subgenotypes in China [27,41,42]. However, BVDV subgenotype 1a was not isolated from cattle in China. Moreover, pathogenesis of above all strains was seldom reported.

In this study, one virus was isolated from nasal swabs of cattle using MDBK cell cultures, and identified as a BVDV isolate by the virus neutralization test, reverse transcriptase-polymerase chain reaction (RT-PCR) method and immunofluorescence assay. To investigate the genetic subgroup of the strain, the 5′UTR gene of the virus was sequenced and compared with other 13 reference BVDV strains by phylogenetic analysis. The pathogenesis of the virus was evaluated by intranasally inoculating to four susceptible calves to assess the potential endemic risk to the cattle herd in China.

## Results

### Virus isolation

**Figure 1:**
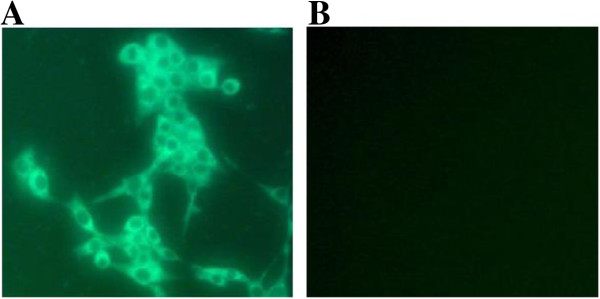
**Immunofluorescence test result on MDBK cells. (A)** BVDV was detected by indirect immunofluorescence assay. Specific fluorescene in form of granules distribution all over the cytoplasm was observed in the infected MDBK cells. **(B)** The control cells.

A virus isolate was isolated from the nasal swab samples after two blind passages on MDBK cells. The MDBK cells inoculated with nasal swab samples developed obvious cytopathic effects (CPE) as cells appeared aggregated and formed net-like in the monolayers within 48 hours of incubation. The non-inoculated control cells did not show the CPE. Specific fluorescent staining showed the appearance in form of granules distribution all over the cytoplasm in the infected MDBK cells, when an indirect immunofluorescence test was conducted using BVDV1 type-specific monoclonal antibodies (E2/gp53 IgG2a Isotype, VMRD, USA). The control cells did not show any fluorescence (Figure 1).

In virus neutralization assay using BVDV anti-sera, results revealed that the inoculated MDBK cells did not show CPE after mix of the virus fluids with BVDV1 positive serum, but cells inoculated with virus fluid only developed CPE. These results indicate that the virus isolate was a BVDV 1 strain.

### Amplification, sequencing, and analysis of 5′UTR

The fragment of 288 bp of 5′UTR gene was amplified from the isolated virus by RT-PCR. The amplified product was purified, cloned and sequenced. The sequence of the isolated BVDV1 strain was compared to the reference sequences using BLAST program. Result revealed that the isolated BVDV 1 strain was similar with the BVDV strains Singer, with sequence identity about 95%. The sequence of 5′UTR has been deposited in GenBank with the accession No.JX878887.

### Phylogenetic analysis

**Figure 2:**
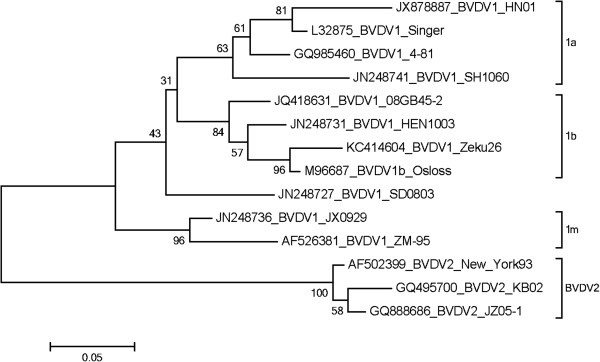
**Phylogenetic tree analysis based the 5′UTR.** The tree was created using the nucleotide sequences of the 13 BVDV strains retrieved from GenBank. The phylogenetic tree was prepared using the Neighbor-Joining method.

Phylogenetic analysis based on 5′UTR revealed the strain HN01 was closed to BVDV1a Singer (L32875). The homology for nucleotide of 5′UTR is 95.3%, and with the strain SH1060 (JN248741) was 89.2%. This result indicates that the newly isolated BVDV strain, HN01, belongs to subgroup BVDV 1a of genotype BVDV1 (Figure 2).

### Experimental infection

**Figure 3:**
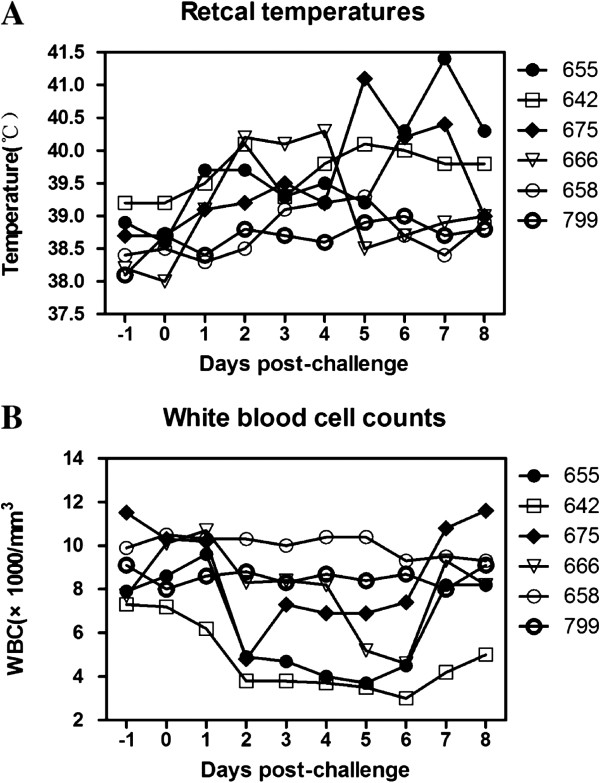
**Experimental infection intranasal inoculating with the HN01 stain. (A)** Elevated rectal temperatures. **(B)** Decrease of white blood cell counts.

Clinical signs were scored based on clinical severity, included depression, loss of appetite, nasal discharge, distress, excessive salivation, and elevated rectal temperatures. Two of the four BVDV infected calves developed significant clinical signs with nasal discharge and depression beginning at 2 days post-challenge (dpi), and subsequently 3 of four infected calves showed asthma and excessive salivation. Calves in the control group had no clinical signs during the experiment course. The rectal temperature of cattle #655 was over 40°C from 6 dpi to 8 dpi with the highest temperature of 41.4°C. The rectal temperature of cattle #642 was over 40°C at 2, 5 and 6 dpi with the highest recorded temperature of 40.1°C. The rectal temperature of animal #675 was over 40°C from 5 to 7 dpi with the highest temperature of 41.1°C. The rectal temperature of cattle #666 was elevated to 40°C from 2 to 4 dpi with the higest temperature of 40.3°C (Figure 3A).

WBC counts of challenged calves started decreasing from day 2 post-challenge. The WBC counts of 655# dropped significantly from 8.6 (1000 per mm^3^) at day 0 to 3.6 (1000 per mm^3^) at 4 dpi, a 58% reduction to the initial number. 642# decreased from 7.2 (1000 per mm^3^) (0 day) to 3.0 (1000 per mm^3^) (6 dpi), a 58% reduction to the initial number. The cattle of 675# decreased from 10.3 (1000 per mm^3^) (0 day) to 4.8 (1000 per mm^3^) (2 dpi), a 53% reduction to the initial number. The cattle of 666# decreased from 10.1 (1000 per mm^3^) (0 day) to 4.6 (1000 per mm^3^) (6 dpi), a 54.5% reduction to the initial number. Average greatest decline of WBC counts among the control animal group was less than 20%, which were significantly less (P < 0.05) than those of the challenge group (Figure 3B).

Viral detection was positive from 2 dpi to 8dpi in nasal samples. Viral shedding was detected by virus isolating in MDBK cells from nasal swabs. Two infected cattle had viral shedding as early as 2 dpi. The long shedding period reached to 8 days after inoculation, and the highest shedding peak was reached at 4-8 dpi.

**Figure 4:**
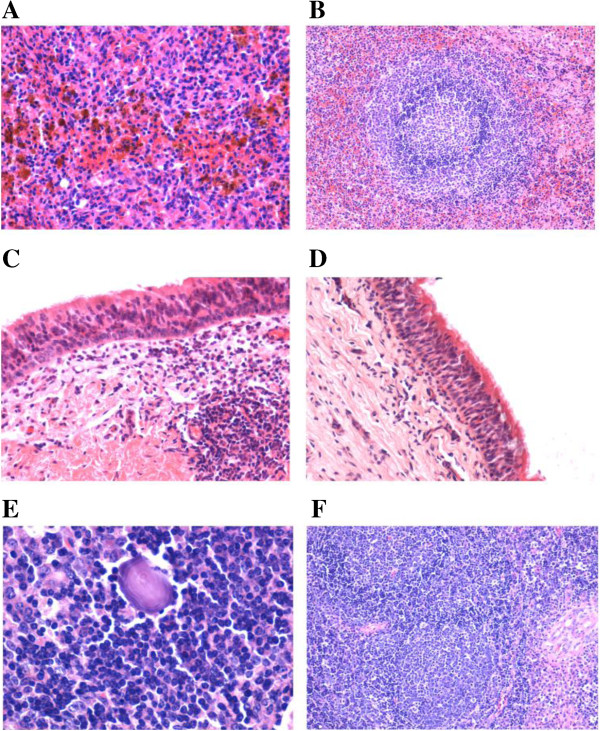
**Histopathologic changes of spleen, trachea and mesenteric lymph nodes. (A)** Red pulp of the spleen hemorrhage, hemosiderin macrophages increased significantly. **(B)** The control was normal. **(C)** Some mucosal epithelium of trachea swollen; neutrophil and plasma cells infiltrated in the lamina propria; lymphocytes proliferated; lymphocytic follicular increased. **(D)** The control was normal. **(E)** Macrophages and reticular cells proliferated and increased. **(F)** The control was normal.

Two randomly chosen calves (#675 and #642) and one mock calf (#799) were euthanized at 8 dpi for histopathology analysis. Gross pathological findings included hemorrhages in the spleen and mesenteric lymph nodes. Histopathologic changes included thymic and spleen atrophy, the lymph node was lymphadenopathy and hemorrhage, and lymphoid depletion of the Peyer′s patches in all the infected calves (Figure 4). All of the samples from the control cattle did not show pathological changes.

## Discussion

This study was the first report on BVDV 1a isolated from cattle in China, and the pathogenesis of the isolate was tested by experimental infection. Similar virus at the genetic subtype was detected in pigs in China [43]. Identification of BVDV 1a in cattle and swine seemed that the virus have evolved well to replicate in different species. To our knowledge, the diversity of the genotype is a dominant character of the BVDV. Phylogenetic analysis is useful for molecular epidemiological studies and vaccine research.as well as tracing the origin of newly introduced viruses [19,44-46]. The 5′UTR analysis is commonly used to classify the genotypes of BVDV strains. To investigate the molecular feature of the strain, 13 reference sequences (Table 1) in China, USA and Korea field isolates were used to phylogenetic analysis. A high genetically identity with the BVDV1a strain was detected among all the reference sequences. Recently, a BVDV 1a strain named SH1060 was isolate from pigs in Shanghai, China [43], but the virus only has the 89.2% homology of 5′UTR with the HN01 reported in this study. This may suggest that the virus have the great variation in evolution between the different kind species.

**Table 1:** Reference sequences from GenBank

**S. No**	**Isolate name**	**Accession number in GenBank**	**Country**
1	Singer	L32875	USA
2	Osloss	M96687	USA
3	New_York93	AF502399	USA
4	HN01	JX878887	China
5	ZM-95	AF526381	China
6	JX0929	JN248736	China
7	SD0803	JN248727	China
8	HEN1003	JN248731	China
9	Zeku26	KC414604	China
10	JZ05-1	GQ888686	China
11	SH1060	JN248741	China
12	KB02	GQ495700	Korea
13	08GB45-2	JQ418631	Korea
14	4-81	GQ985460	Korea

The subgroup of BVDV might be correlated with the geographical origin [47]. Previous study indicated that the BVDV 1a subtype was predominant and widespread in neighboring countries, such as Korea and Japan [47,48]. In China and India, BVDV 1b was the predominant subgenotypes [27,49]. Hence, the isolated BVDV1a strain in this study may be originated from the import of infected livestock. The findings of the current study also suggest a possible role of BVDV in the etiology of diarrhea and bovine respiratory disease. In addition, the presence of BVDV could represent a clinical threat to diary and cattle industry in China.

In this study, calves infected strain HN01 of BVDV1a showed the mild clinical signs, high rectal temperature and lymphopenia, which suggested that the strain had the moderate virulence to cattle. Previous studies on the pathogenesis of BVDV1 and BVDV2 demonstrated that the low virulent strains were characterized with a subclinical course, a mild short-term pyrexia, rectal temperatures between 39.2°C and 40.0°C for 1-2 days, and a drop in circulating lymphocytes ranging between 20% and 40% [50]. In contrast, clinical signs infected with a high virulent strain were severe with a longer term and more severe pyrexia, temperatures above 40°C, ranging upwards to 41.7°C for 3 or more days, decreases of greater than 40% in circulating lymphocytes and decreases of greater than 40% in platelets. Death losses ranging between 20% and 50% have been observed in field outbreaks associated with highly virulent BVDV2 strains [36,39,51]. Although the experimental animals showed over 40°C for 2 days, over 40% WBC drop, the virus was categorized as moderate virulence due to the lack of severe clinical signs. This study demonstrated that strain HN01 was an alternate challenge strain in addition to the currently available strain NY-1 for vaccine efficacy test from the USDA [39]. Furthermore, much of the research on BVDV virulence demonstrated that the clinical signs, such as depression, febrile response and excessive lacrimation were more reproducible than hemorrhage or diarrhea. It may be suggested that low virulence strains were more dominant in nature [52-54]. Despite the lack of severe clinical signs, infection with BVDV may transiently compromise the immune system and thus possibly predisposes cattle to secondary infections [52]. Measures such as vaccination should be taken to control BVDV1 infection.

## Conclusions

In this study, a BVDV strain genotype 1a was firstly isolated from cattle herd in China. The study carried out a molecular epidemiology and pathogenesis on the strain. Calves inoculated with the isolate developed clinical signs, including high temperature, depression, cough and nasal discharge. The results confirmed the existence of the BVDV type 1a in China, and it may be helpful in preventing the BVD in China and a challenge virus strain for efficacy evaluations of vaccines.

## Materials and methods

### Samples and virus isolation

Twenty nasal swabs and blood samples were collected from the cattle that showed mild respiratory clinical signs, such as nasal discharge and cough in Henan province, China in 2010. The nasal swab samples were collected and put into a tube containing 2 ml DMEM (HyClone, USA) supplemented with 10% horse serum (Hyclone, USA), 150 μg/ml gentamicin sulfate (Sigma, USA), 7.5 μg/ml fungizone (Sigma, USA), and Streptomycin at 100 μg/ml. Three milliliter (3 ml) blood samples were collected using an EDTA vacuum blood tube from the jugular vein. All the samples were kept at 2-8°C and quickly transferred to the laboratory. Nasal swabs were inoculated into MDBK cell monolayers in 24-well tissue culture plates for virus isolation. Briefly, following centrifugation at 1500 rpm for 10 min, the samples were filtered through 0.45 μm membrane (Sigma, USA) and then inoculated onto the MDBK cell monolayer cultured in 0.5 ml DMEM (HyClone, USA) supplemented with 6% horse serum (Hyclone, USA) in 24-well cell culture plates, and incubated at 37°C, with 5% CO_2_ for 2 hours. Then, the supernatants were discarded, and plates were rinsed twice with PBS (pH7.2, 0.01 mol/L), and 1 ml DMEM (HyClone, USA), with 3.5% horse serum was added. The infected-MDBK cell plates were checked daily and appearance of cytopathic effects (CPE) was observed and recorded. If the CPE was not found, the cultures were frozen and thawed twice and the clarified supernatant was passaged three times in MDBK cells. Un-infected MDBK cultures were included as negative controls and MDBK cells inoculated with BVDV NM01 strain, which was previously isolated and identified by our laboratory, was used as positive control. After 3-4 days of incubation at 37°C, with 5% CO_2_ supply, the virus was confirmed by immunofluorescence on cell monolayers as described below.

### Virus neutralization test

To identify the isolated virus, virus neutralization (VN) test was conducted using antisera raised in cattle against BVDV1a NM01, a vaccine strain in our laboratory. Briefly, virus fluids were mixed with the equal volume hyperimmune antisera (antibody VN titer ≥ 1∶64) and incubated at 37°C for one hour, and then the mixture was transferred into MDBK cell cultures in the 96-well tissue culture plates. After 4 days of incubation at 37°C, with 5% CO_2_ supply, the plates were observed for CPE to determine if the virus isolate was neutralized by the BVDV antisera. The antisera positive control and negative control were also included in this test.

### Immunofluorescence assay

To detect the BVDV in the infected-MDBK culture, an immunofluorescent assay (IFA) was conducted as follows. Briefly, cell lysate was added to each of four wells of a 96-well MDBK tissue culture plate. Positive virus (NM01 strain) and DMEM media (negative control) were also added to 4 wells respectively in the test. Then plates were incubated for 4 days at 37°C, with 5% CO_2_ supply. Then, the supernatants were dumped and plates were fixed with the mixture of 80% acetone and 20% methyl alcohol; the fixed plates were incubated with monoclonal antibody specific to anti-BVDV1 (E2/gp53 IgG2a Isotype, VMRD, USA). Finally, the rabbit anti-mouse fluorescein isothiocyanate (FITC)-conjugated immunoglobulin G (IgG) (Sigma, USA) was added to the plates, then incubated in a 37°C humid box for an hour. The plates were examined for fluorescence under a fluorescent microscope (Zeiss Axioskop-40, Germany).

### PCR amplification and sequencing

Primers (Forward: 5′ATGCCCTTAGTAGGACTAGCA3′; Reverse: 5′TCAACTCCATGTGCCATGTAC3′) for 5′UTR gene sequence of BVDV were designed according to the BVDV strain Singer (GenBank accession number L32875) and strain NADL (GenBank accession number M31182). Total BVDV RNA was extracted from infected MDBK cell fluids using Trizol reagent (Invitrogen, China) according to the manufacturer′s instructions. The reverse transcription polymerase chain reaction (RT-PCR) was carried out using 5 μl of RNA extraction samples and 1 μl of BVDV reverse primer as reverse transcription primer in a 0.2 ml tube for 5 min at 70°C; and the tube was added with 1 μl (200U) M-MLV reverse transcriptase RNase inhibitor and 2 μl RNAse-free water, and then was incubated for one hour at 42°C. The amplification of cDNA by PCR was carried out in a total volume of 50 μl solution containing 1 μl cDNA, 5 μl 10 × Buffer, 3 μl 2.5 mmol/L dNTP, 0.5 μl each primer, 0.5 μl Platinum Pfx DNA polymerase and 39.5 μl sterile water. Amplification was performed as follows: 95°C for 5 min, and then submitted to 30 cycles of amplification. The conditions for the amplification were 1 min at 94°C, 1 min at 55°C, and 1 min at 72°C, then with a final extension at 72°C for 10 min. The PCR products were detected by electrophoresis through a 1% agarose gel stained with ethydium bromide and visualized under UV light. The target fragment of PCR products were purified and cloned into pMD18-T vector (TaKaRa, Japan), and then transfected into E.coli DH5α cells. The positive clones were selected for sequencing by Shanghai Invitrogen Biotechnology Co., Ltd.

### Sequences analysis

The sequence analysis was performed by computer software DNASTAR (Madison, Wis USA) and MEGA5.1. The 5′UTR gene sequence of the virus isolate was evaluated for further sub-genotype classification and compared to 13 BVDV reference sequences (Table 1) from NCBI database information to develop a phylogenetic tree. Percent identities and divergences were calculated using Neighbor-Joining method of Mega5.1 software.

### Experimentally infection

#### Calves and housing

Six 4-month old calves were obtained from a calf farm in Inner Mongolian, China. All animals were negative to BVDV and apparently healthy, presenting no signs of depression, cough or other health disorders. The selected animals were transported to an animal facility in Inner Mongolian. All animal experiments were approved by the Institutional Animal Care and Use Committee of Jilin University.

### Virus infection and temperature recording

Three days prior to challenge, all animals were transferred to a bio-level 3 safety facility. Four calves were intranasally inoculated with 6 ml of cell culture medium containing 10^6.8^TCID_50_ per milliliter of the HN01 strain, and other two calves were inoculated with the cell culture medium to serve as the controls. All the culture medium were demonstrated free of adventitious pathogens. The challenge procedure was performed by spraying 3 ml of virus samples into each nostril, using an atomizer, and then the calves were monitored for 8 days. The temperature was taken two times at the same time every day by investigators who were unaware of the treatment codes in each study. Three randomly chosen calves were euthanized at 8 dpi for histopathology analysis.

### Clinical assessment

Calves were clinically observed daily from 1 to 8 days post-challenge. Clinical signs including depression, cough, asthma, and other respiratory disease were recorded and scored using a scale of 0 – 3 (0 = absence; 1 = mild; 2 = moderate; 3 = severe).

### Sample collection

White blood cell (WBC) counts were conducted from 2 days pre-challenge through 8 days post-challenge and white blood cell (WBC) counts were conducted by a Vetscan HM5 veterinary hematology system (Abaxis, USA). Deep nasal swab specimens were collected at 1 day prior to challenge through 8 days post-challenge. After collection, swabs were placed into a tube containing 3 ml of transport medium, consisting of DMEM (HyClone, USA) supplemented with 10% horse serum (Hyclone, USA), 150 μg/ml gentamicin sulfate (Sigma, USA), 7.5 μg/ml fungizone and streptomycin at 100 μg/ml. The samples were kept at 2-8°C and quickly transferred to the laboratory. Upon arriving in laboratory, all swab specimens were stored at -70°C or below until they were cultured for virus isolation.

### Histopathologic study

On days 8 dpi, two calves in the infected group and one in the control group were euthanized; tissue samples of liver, spleen, lung, heart, kidney, intestine, mandibular lymph node, and mesenteric lymph node were collected and fixed in 10% buffered formalin for histopathologic analysis in Inner Mongolia agricultural university.

## Competing interests

The authors declare that they have no competing interests.

## Authors’ contributions

WW participated in the design and conducted the majority of the experiments in the study and drafted the manuscript. XS and QT participated in the molecular genetic studies and the sequence alignment. YW and YJ took the animal experiment and collected samples. MX was performed the statistical analysis. WX and HW conceived of the study, and participated in its design. All authors read and approved the final manuscript.
